# The Immune Landscapes of Polypoid and Nonpolypoid Precancerous Colorectal Lesions

**DOI:** 10.1371/journal.pone.0159373

**Published:** 2016-07-21

**Authors:** Antonella Maglietta, Rosalia Maglietta, Teresa Staiano, Ramona Bertoni, Nicola Ancona, Giancarlo Marra, Leonardo Resta

**Affiliations:** 1 Department of Emergency and Organ Transplantation, University of Bari, Bari, Italy; 2 Institute of Intelligent Systems for Automation, National Research Council, CNR-ISSIA, Bari, Italy; 3 Endoscopy and Gastroenterology Unit, Hospital of Cremona, Cremona, Italy; 4 Department of Pathology, Hospital of Cremona, Cremona, Italy; 5 Institute of Molecular Cancer Research, University of Zurich, Zurich, Switzerland; Mie University Graduate School of Medicine, JAPAN

## Abstract

Little is known about the immunoediting process in precancerous lesions. We explored this aspect of benign colorectal adenomas with a descriptive analysis of the immune pathways and immune cells whose regulation is linked to the morphology and size of these lesions. Two series of polypoid and nonpolypoid colorectal adenomas were used in this study: 1) 84 samples (42 lesions, each with matched samples of normal mucosa) whose gene expression data were used to quantify the tumor morphology- and size-related dysregulation of immune pathways collected in the Molecular Signature Database, using Gene Set Enrichment Analysis; 2) 40 other lesions examined with immunohistochemistry to quantify the presence of immune cells in the stromal compartment. In the analysis of transcriptomic data, 429 immune pathways displayed significant differential regulation in neoplasms of different morphology and size. Most pathways were significantly upregulated or downregulated in polypoid lesions versus nonpolypoid lesions (regardless of size). Differential pathway regulation associated with lesion size was observed only in polypoid neoplasms. These findings were mirrored by tissue immunostaining with CD4, CD8, FOXP3, MHC-I, CD68, and CD163 antibodies: stromal immune cell counts (mainly T lymphocytes and macrophages) were significantly higher in polypoid lesions. Certain markers displayed significant size-related differences regardless of lesion morphology. Multivariate analysis of variance showed that the marker panel clearly discriminated between precancerous lesions of different morphologies and sizes. Statistical analysis of immunostained cell counts fully support the results of the transcriptomic data analysis: the density of infiltration of most immune cells in the stroma of polypoid precancerous lesions was significantly higher than that observed in nonpolypoid lesions. Large neoplasms also have more immune cells in their stroma than small lesions. Immunoediting in precancerous colorectal tumors may vary with lesion morphology and stage of development, and this variability could influence a given lesion’s trajectory to cancer.

## Introduction

The immune system plays a Janus-like role in the development and progression of cancer, exerting tumor-promoting and tumor-suppressive effects. This duality is the basis of the process known as *immunoediting*, whereby the immune system shapes (or edits) the evolution of tumorigenesis, qualitatively and quantitatively [1]. Outcomes envisioned by this model range from 1) the outright *elimination* of tiny, nascent neoplastic lesions by joint intervention of the innate and adaptive immune systems; 2) a state of *equilibrium* between tumor cells and host, during which the adaptive immune system checks frank outgrowth and maintains a state mildly conducive to slow neoplastic proliferation; and 3) *tumor escape*, characterized by the emergence, under selective immune pressure, of decreasingly immunogenic neoplastic cell populations and a state of frank immunosuppression [1]. Recognition and ongoing characterization of these phenomena have prompted attempts to combat or control cancer by pharmaceutical modulation of the immunoediting process (e.g., enhancing the presence of immune effector cells with anti-tumor activities, inhibiting or eliminating molecular and cellular mediators of cancer-induced immunosuppression). The mechanisms underlying the equilibrium phase are of particular interest. This state of relative dormancy is typical of the benign, preinvasive stages of tumorigenesis, and it can keep malignancy at bay for years—up to two decades in some cases of colorectal neoplasia [2, 3, 4]. Prolongation or durable recapitulation of this state is thus regarded as a potentially achievable end point of immunotherapy [1]. The incidence of precancerous colorectal lesions and/or of “interval” cancers (those occurring before the follow-up colonoscopy) can be reduced by chemopreventive interventions with aspirin or other nonsteroidal anti-inflammatory drugs, which alter the tumor microenvironment, rendering it more conducive to the elimination of nascent tumors or the arrest or suppression of the growth of larger lesions [5, 6].

Little is known about the mechanisms underpinning the equilibrium phase in colorectal tumorigenesis. The immune landscape of epithelial tumors is highly complex, and the characteristics (cell types, extension, functionality) of intralesional immune-cell populations vary with the cell-type origin of the tumor and the site where it develops. In large bowel neoplasms, for example, the superficial layer of epithelial cells is the only barrier between the tumor stroma and the bacterial flora of the gut. The composition of the immune infiltrate in these tumors is thus likely to reflect its dual nature, as a response to immunogenic epithelial tumor-cell antigens and to any microbial products that pass through the neoplastic epithelium.

Immune cell infiltrates in invasive colorectal cancers have been well-characterized [7–21], but less is known about the immunomes of precancerous colorectal tumors [22–28]. The present study was an attempt to close that gap.

Precancerous colorectal lesions vary widely in morphology, size, and histology. Their gross appearance at endoscopy is broadly classified as “polypoid” or “nonpolypoid” [29]. The former tumors protrude into the gut lumen and are attached to the mucosa with a pedicle or stalk (pedunculated lesions, type *Ip*) or with a shorter, broader base (sessile lesions, type *Is*). Nonpolypoid lesions are still erroneously described as “flat” by many authors, although most are actually slightly elevated (< 2.5 mm above the mucosal surface; type *IIa*). Those that are truly flat or slightly depressed are rare (reviewed in [4]). In both morphological classes, increasing size reflect the lesion’s advancement on the road to cancer [30]. Most precancerous colorectal lesions—polypoid or nonpolypoid—are *adenomas*, and their degree of dysplasia increases with their size. Nonadenomatous precancerous lesions, the so-called sessile serrated adenomas/polyps, are much less common and rarely display epithelial dysplasia [31].

For years, the prevalence of nonpolypoid colorectal neoplasms in Western countries has been markedly underestimated. Today, however, thanks to improved awareness and training of colonoscopists and to advances in endoscopic technology, these lesions represent up to ~40% of the precancerous lesions identified during colonoscopy, particularly those found in the proximal colon [32, 33]. Some investigators have suggested that these tumors progress more rapidly to cancer than polypoid lesions [34], but in other studies the frequencies of carcinoma in nonpolypoid lesions resembled [35,36] or was lower than [33] that observed in polypoid lesions. The discrepancies are largely due to inconsistencies between studies in the use of endoscopic and histologic classifications of precancerous colorectal tumors (reviewed in [37]). At the molecular level (i.e., gene expression, genetic and epigenetic alterations), however, unequivocal differences have been documented between the epithelia of nonpolypoid and polypoid lesions [37, 38]. Therefore, these two categories of lesions can reasonably be expected to differ in terms of their tumor microenvironments, immune-cell infiltrates in particular.

In this study, we used gene set enrichment analysis (GSEA) [39] to explore a gene expression data set derived from endoscopic biopsies of polypoid and nonpolypoid colorectal adenomas. Our aim was to identify immune pathway regulation profiles related to lesion morphology and/or size. The results were validated by immunohistochemical analysis of formalin-fixed, paraffin-embedded (FFPE) sections from a second series of precancerous colorectal tissues.

## Results

### Gene set enrichment analysis

To identify biological pathways that might play key roles in the development of precancerous lesions in the colorectum, we used gene set enrichment analysis (GSEA) (Subramanian et al. 2005) with the Molecular Signatures Database (MSigDB) to explore the transcriptomic profiles of 42 precancerous colorectal neoplasms: 17 were polypoid lesions (9 small, 8 large), and 25 were nonpolypoid (10 small, 15 large) (see Methods for details). The original data had been obtained with microarray analysis of endoscopic mucosal biopsies containing neoplastic epithelium as well as stroma. The results were normalized to matched samples of non-neoplastic colorectal mucosa.

GSEA of our transcriptomic data using all the collections in the MSigDB revealed enrichment for immune-related pathways in the set of genes differentially expressed in the two morphologic classes of lesions. We therefore restricted our analysis to the C7 collection, which included 1910 gene sets representing various cell states and perturbations within the immune system. Five group comparisons were performed: 1. *polypoid vs*. *nonpolypoid (all lesion sizes)*; 2. *polypoid vs*. *nonpolypoid (small lesions only)*; 3. *polypoid vs*. *nonpolypoid (large lesions only)*; 4. *small vs*. *large (polypoid lesions only)*; 5. *small vs*. *large (nonpolypoid lesions only)*. Diameter, the standard measurement of lesion size in endoscopic reports, was analyzed as an index of the precancerous lesions’ malignant potential [30].

**Fig 1:**
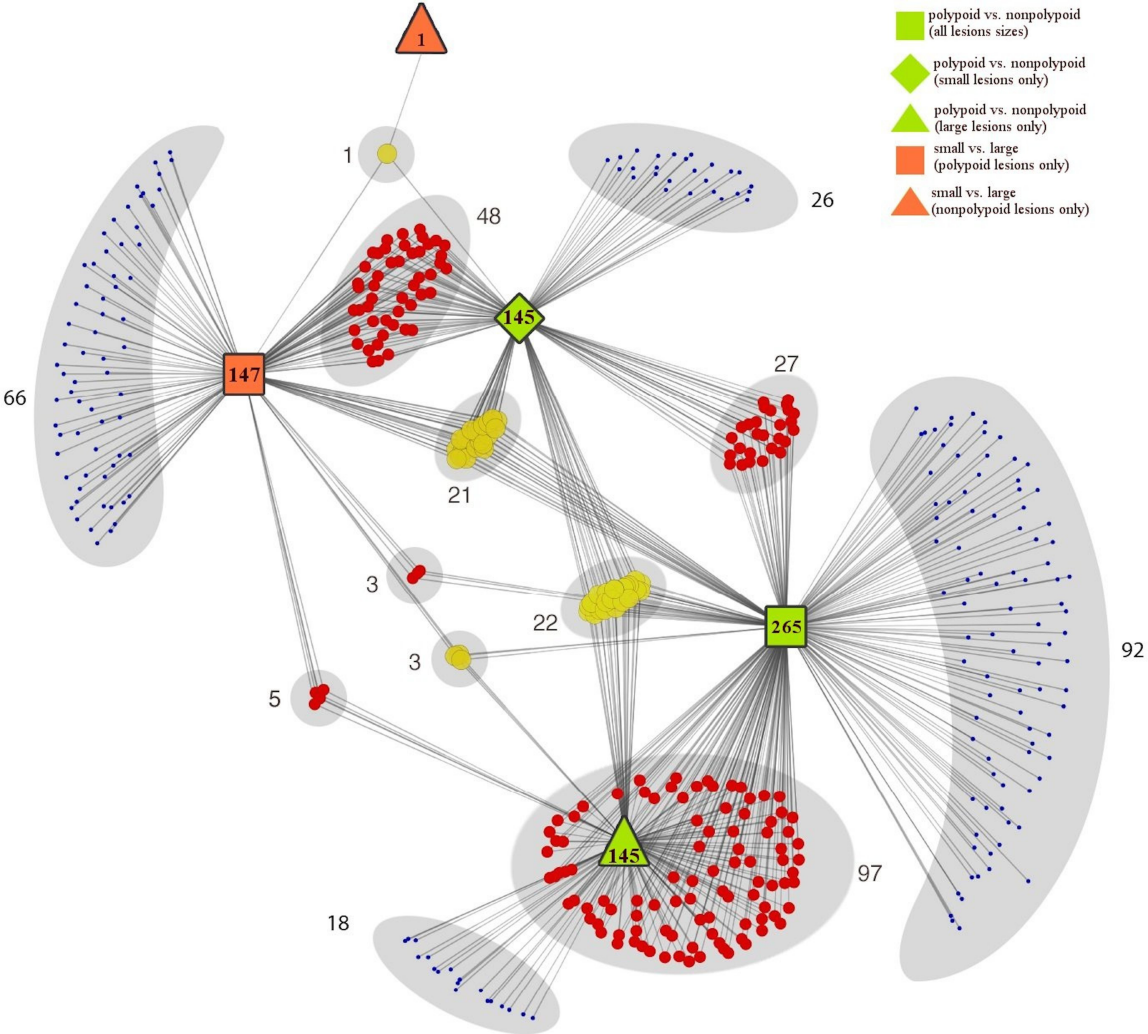
Distribution of immune pathways displaying significant dysregulation in GSEA analysis of the five group comparisons. Colored circles represent 429 pathways that exhibited significant dysregulation (p-value < 0.05) in the GSEA analysis of one (blue), two (red), or three (yellow) of the five group comparisons: 1) polypoid vs. nonpolypoid (all lesions sizes, green squares); 2) polypoid vs. nonpolypoid (small lesions only, green diamonds); 3) polypoid vs. nonpolypoid (large lesions only, green triangles.); 4) small vs. large (polypoid lesions only, orange squares.); 5) small vs. large (nonpolypoid lesions only, orange triangle). The complete list of differentially regulated pathways with their p-values is reported in **S1 Table**.

**Fig 1**and **S1 Table** show the 429 immune pathways that displayed significant differential regulation (p-values < 0.05) in the GSEA of one or more of the five group comparisons. Two hundred sixty-five pathways were significantly upregulated (n = 219) or downregulated (n = 46) in polypoid lesions relative to their nonpolypoid counterparts (all sizes). This morphology-associated difference was equally striking within both size classes: 145 immune pathways were upregulated (n = 35) or downregulated (n = 110) in small polypoid lesions compared with small nonpolypoid lesions, and the same number were differentially regulated (144 upregulations, 1 downregulation) in large polypoid lesions relative to large lesions with nonpolypoid morphologies. Lesion size was also associated with differential regulation of numerous immune pathways in the polypoid lesions (4 upregulated pathways and 143 downregulated pathways in small vs. large polypoid tumors). In the nonpolypoid group, only 1 pathway displayed downregulation in small lesions compared with the large lesions. Collectively, these findings strongly suggest that the immunologic signatures of polypoid adenomas are markedly different from those of their nonpolypoid counterparts, independently of their size. However, the signature of polypoid lesions also appears to change appreciably with lesion size.

### Immunohistochemistry studies

The results reported above were based on transcriptomic analysis of composite tissue biopsies with epithelial to stromal cell ratios of around 2:1. Assuming they are valid, one would expect to see similar trends emerge from analyses restricted to the stromal immune-cell landscape of precancerous colorectal lesions. We therefore examined a second, archival series of 40 colorectal adenomas (see Methods for details)—19 polypoid lesions (10 small, 9 large), 21 nonpolypoid lesions (10 small, 11 large). Immunohistochemistry was used for this purpose since it preserves tissue architecture and allows quantification of cellularity in specific tumor regions. These lesions were subjected to the same five group comparisons used for the pathway analysis.

**Fig 2:**
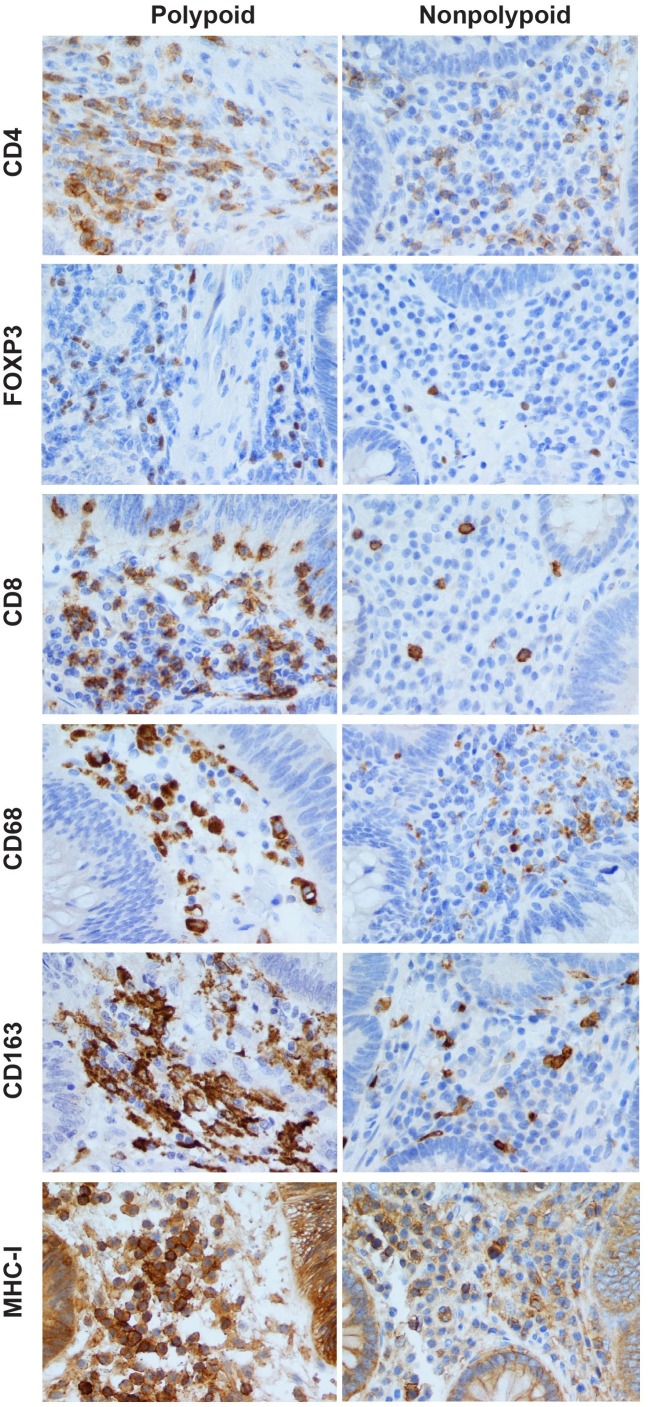
Immunohistochemical assessment of stromal immune cell infiltrates in polypoid and nonpolypoid colorectal adenomas. CD4+, FOXP3+, CD8+, CD68+, CD163+, and MHC-I+ immune cell densities (i.e., number of positively-stained cells per unit area; see Methods) in the stroma of polypoid adenomas were consistently higher than those observed in nonpolypoid lesions. Intraepithelial CD8+ (cytotoxic) T cells are also seen in the epithelial compartments of both types of tumor. In calculating CD68+ and CD163+ macrophage densities, we counted only nuclei surrounded by CD68+ or CD163+ granulations and excluded elongated or fragmented cells with granular cytoplasmic positivity. MHC-I labelling (granular, compact, or membranous) was observed in a variety of stromal cells. MHC-I+ epithelial cells were also present in both types of tumor, but their presence was not quantified in this study. Magnification for all panels: 400X.

The immune cell populations most highly represented in the pathways shown in **S1 Table** were T-lymphocytes and macrophages (~50% and ~6% of the pathways, respectively); B-cell, NK-cell, and other immune cell signatures were all substantially less common. Serial sections of FFPE tissues were thus stained with antibodies against the T-lymphocyte markers CD4, CD8, and FOXP3 and the macrophage markers CD68 and CD163 (see Methods). Antibody against a sixth marker, MHC-I, was used to obtain a general, nonspecific count of stromal cells, including certain immune cells that were not covered by the other markers we used (e.g., dendritic cells, natural killer cells) (see Discussion). **Fig 2**shows representative findings for each marker in the stroma of polypoid and nonpolypoid colorectal adenomas.

**Fig 3:**
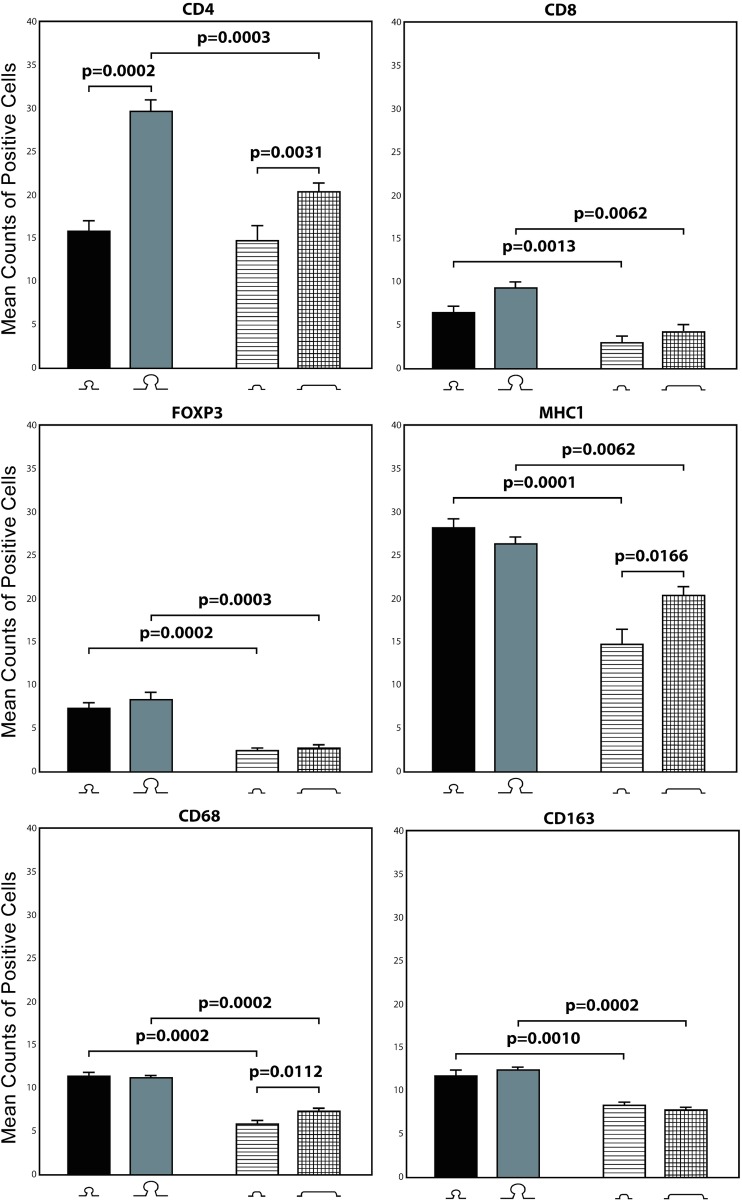
Immunohistochemical morphometric analysis of stromal immune cells. Bars represent mean (SE) CD4+, CD8+, FOXP3+, MHC-I+, CD68+ and CD163+ cell counts for the following groups (from left to right): small polypoid lesions, large polypoid lesions, small nonpolypoid lesions, large nonpolypoid lesions. Horizontal bars with P values indicate significant differences between groups (Mann-Whitney test) (see **Table 1**for details).

As shown in **Fig 3**and **Table 1,** for each marker the number of positively labelled stromal immune cells was significantly higher in polypoid than nonpolypoid lesions. These differences were generally independent of lesion size, i.e., the increased presence in polypoid adenomas was still significant when analysis was confined exclusively to the subset of small or large tumors. CD4+ cells were the exception: the higher cell counts in polypoid lesions vs. nonpolypoid were significant only in large lesions. As for size-related differences, CD4+ cell counts were also significantly higher in large polypoid lesions than in small lesions with the same morphology. In nonpolypoid lesions, CD4+, MHC-I+, and CD68+ cells were significantly more common in those that were large. (CD8+ T cells were also present within the neoplastic epithelium, but their numbers in this compartment showed no significant morphology- or size-related variations, and they were thus excluded from the statistical analysis.)

**Table 1:** Significant differences between mean labelled immune cell counts in the five group comparisons.

Group Comparisons	Group with higher counts	CD4+	CD8+	FOXP3+	MHC-I+	CD68+	CD163+
polypoid vs. nonpolypoid (all sizes)	polypoid		10^-5	10^-7	10^-6	10^-8	10^-7
polypoid vs. nonpolypoid (small lesions only)	polypoid		0.0013	0.0002	0.0001	0.0002	0.001
polypoid vs. nonpolypoid (large lesions only)	polypoid	0.0003	0.0062	0.0003	0.0062	0.0002	0.0002
small vs. large (polypoid lesions only)	large	0.0002					
small vs. large (nonpolypoid lesions only)	large	0.0031			0.0166	0.0112	

P-values, (Mann-Whitney test) are shown for each difference; statistical significance was set at p-value < 0.05. The direction of mean counts difference is shown for each marker in the five group comparisons.

**Fig 4:**
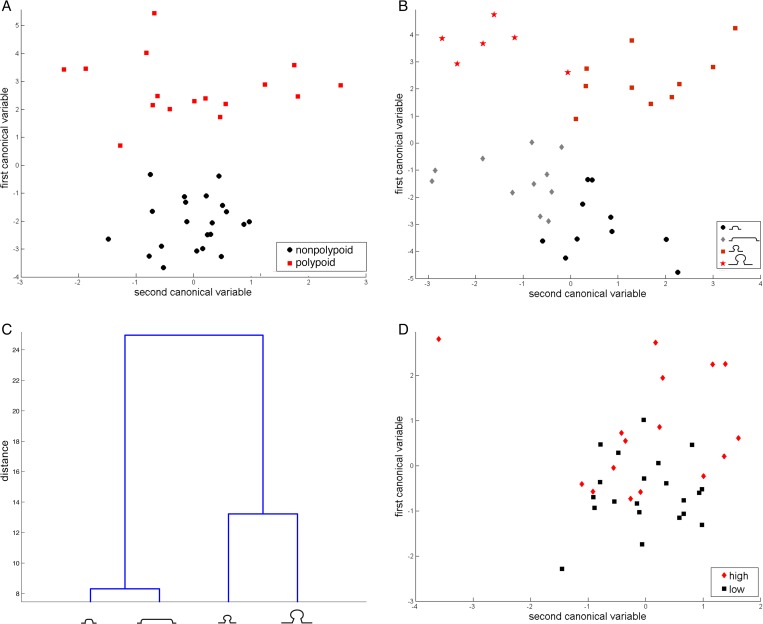
Multivariate analysis of polypoid and nonpolypoid lesions (small and/or large) using MANOVA test. A) Scatter plot of the first two canonical variables for all polypoid (red squares) and all nonpolypoid lesions (black circles); B) scatter plot of the first two canonical variables for small polypoid lesions (red squares), large polypoid lesions (red stars), small nonpolypoid lesions (black circles), large nonpolypoid lesions (gray diamonds); C) dendogram plot of group mean clusters; D) scatter plot of the first two canonical variables for all adenomas with high-grade dysplasia (red diamonds) and all adenomas with low-grade dysplasia (black squares), regardless of morphology and size. In the scatter plots, each dot represents one tissue sample.

One-way multivariate analysis of variance (MANOVA) was then used to compare the multivariate means of the lesion groups in the five group comparisons (**Table 2)**. Significant p-values were found in all five group comparisons, indicating that the number of cells labelled with the 6 immune markers we used effectively discriminated between adenomas of different shapes and sizes. When only lesion morphology was considered, the canonical analysis by MANOVA revealed that polypoid lesions were perfectly separated from nonpolypoid lesions along the axis of the first canonical variable (**Fig 4A**). When both morphology and size were considered, small and large lesions were clearly separated along the axis of the second canonical variable in both morphological classes (**Fig 4B**). Indeed, the main intergroup differences were related to lesion morphology and, within each morphology class, to lesion size (**Fig 4C**).

**Table 2:** Multivariate analysis of the immune cell counts in the five group comparisons.

Comparison	p-value
polypoid vs. nonpolypoid (all sizes)	10^**−11**^
polypoid vs. nonpolypoid (small lesions only)	10^**−5**^
polypoid vs. nonpolypoid (large lesions only)	10^**−5**^
small vs. large (polypoid lesions only)	0.001
small vs. large (nonpolypoid lesions only)	10^**−4**^

P-values were computed by MANOVA test.

Because lesion size is positively correlated with the degree of epithelial cell dysplasia in both polypoid and nonpolypoid adenomas [30], we analyzed the possible association between adenoma diameters and stromal immune cell counts. Because the number of samples was small, we compared lesions with high and low degrees of dysplasia only in the following groups of lesions: 1) all 39 lesions (one small, nonpolypoid lesion was excluded because it displayed histologic features of a sessile serrated adenoma/polyp, see Methods); 2) the 19 polypoid lesions; 3) the 20 nonpolypoid lesions; 4) the 19 small (polypoid and nonpolypoid) lesions; 5) the 20 large (polypoid and nonpolypoid) lesions (**Table 3**). The degree of epithelial dysplasia was significantly associated only with stromal counts of CD4+ and CD8+ cells. They were significantly more numerous in lesions displaying high-grade dysplasia (p-values of 0.002 and 0.048, respectively), regardless of morphology or size. In the polypoid lesion group, tumors of all sizes with high-grade dysplasia had significantly higher CD4+ cell counts (p-value = 0.003 vs. polypoid lesions with low-grade dysplasia). In the small lesion subset, tumors with high-grade dysplasia had significantly higher CD8+ cell counts (p-value = 0.026 vs. small lesions with low-degree dysplasia). The difference between tumors with high- and low-grade dysplasia displayed borderline significance in the group composed of all 39 lesions (p-value = 0.049), while the differences observed in the remaining four groups were not statistically significant. This finding suggests that the 6-marker panel does not discriminate well between lesions with high- and low-grade dysplasia. Indeed, scatter plots of these data produced two overlapping clouds of points (**Fig 4D**).

**Table 3:** Significant increases in labelled immune cell counts in lesions with high- vs. low-grade dysplasia.

Comparison	group	CD4	CD8	FOXP3	MHC-I	CD68	CD163
high- vs. low-grade dysplasia	all lesions	0.002	0.048	-	-	-	-
high- vs. low-grade dysplasia	polypoid	0.003	-	-	-	-	-
high- vs. low-grade dysplasia	nonpolypoid	-	-	-	-	-	-
high- vs. low-grade dysplasia	small	-	0.026	-	-	-	-
high- vs. low-grade dysplasia	large	-	-	-	-	-	-

p-values computed by Mann-Whitney test are shown, and the criteria for statistical significance was set at p-value < 0.05.

## Discussion

Our GSEA of the transcriptomes of precancerous colorectal lesions revealed major differences between the immune signatures of tumors with polypoid and nonpolypoid morphologies. Within the polypoid group, significant differences also emerged between small and large lesions. The most striking differences (**S1 Table**) were the focus of our stromal immunohistochemistry studies in a second set of colorectal adenomas. The latter analyses confirmed that T lymphocyte and macrophage cell counts were significantly higher in polypoid lesions (versus nonpolypoid tumors), regardless of size. Significant size-associated differences were also observed for certain immune markers in polypoid or nonpolypoid lesions (**Table 1**). Indeed, the panel clearly discriminated between precancerous lesions of different shapes and sizes (**Table 2**and **Fig 4**). When the variable *epithelial cell dysplasia* was included in the analysis, statistics were applicable to groups with a reasonable number of lesions. The positive association between high-grade dysplasia and immune-cell density in the stroma of adenomas (**Table 3**and **Fig 4**) displayed only borderline significance and thus requires further analysis in a larger series of tissues.

Ours is the first attempt to determine how the composition of immune cell infiltrates in *precancerous* colorectal lesions correlates with their morphology and/or size. Nonetheless, interesting insights can be gained by comparing our findings with those of the few studies that have investigated this phenomenon at a more general level. Among the stromal cell populations we quantified, CD4+ T helper cells were the most abundant in small adenomas (**Fig 3**), and their densities increased significantly with size (1.9- and 1.5-fold in large lesions from the polypoid and nonpolypoid groups, respectively). In colorectal cancer, progression from stage T1 to T4 is reportedly accompanied by a gradual decline in stromal counts of CD3+ cells, which include those that are CD4+ and/or CD8+ [20]. However, stromal CD4+ cell densities in invasive adenocarcinomas of the colon are significantly higher than those found in adenomas, and these cells are even less common in the normal mucosa [25]. These findings point to a progressive increase in the presence of CD4+ cells across the normal mucosa-adenoma-carcinoma sequence. This is fully consistent with our data showing higher CD4+ T-cell densities in larger and more dysplastic adenomas (**Fig 3**). More detailed sub-type level characterization of the CD4+ cell infiltrates is needed to define the biological and clinical significance of this trend: the T helper (Th) 1 and Th2 subsets have opposite effects on tumor growth, and Th1:Th2 ratios can change radically during tumor progression [18, 26].

Regulatory CD4+ T cells (T_reg_) can be reliably and specifically identified and quantified on the basis of FOXP3 expression. Tumor-infiltrating T_reg_ cells dampen the anti-tumor immune response and promote tumor escape by producing immune-suppressive cytokines, adenosine, and prostaglandin E2 [40, 41]. This is consistent with reports of increasing T_reg_ cell densities in colorectal tumors during progression through the precancerous and early cancerous stages [26, 28]. This trend was not reflected in our data: unlike CD4+ cell densities, T_reg_ density did not increase significantly with lesion size. However, their abundance in the polypoid adenoma sections we examined was almost 3 times as high as that observed in the nonpolypoid lesions (**Fig 3**), and this feature might be expected to favor more rapid growth of polypoid tumors. It is important to recall, however, that tissue populations of T_reg_ cells might include phenotypic and functional subsets, and their immunosuppressor activities might ultimately prove to be contextual. In invasive tumors, for example, heavy T_reg_ cell infiltration is associated with a poor prognosis in ovarian cancer patients [42] and with better prognosis and improved overall survival in those with colorectal cancers [10]. Bindea et al. [20] found that colorectal cancer progression from T1 to T4 was accompanied by decreasing rather than increasing T_reg_ cell densities. In the cancers they studied, the immune cells believed to mediate immunosuppression did not appear to exert any major tumor-promoting effects.

Cytotoxic (CD8+) T cells were also significantly more common in the polypoid adenomas we examined (fold change of 2.2 relative to nonpolypoid lesions) (**Fig 3**), and their density did not increase significantly with tumor size. In general, these cells were much less abundant than CD4+ T helper cells in adenomas, with CD8+/CD4+ ratios of 0.4 (small polypoid lesions), 0.3 (large polypoid lesions), and 0.23 (nonpolypoid lesions of all sizes). McLean et al. reported a CD8+/CD4+ ratio of 0.27 in adenomatous polyps in general (there was no differentiation based on morphology) and noted that the CD8+ cell density in these tumors resembled that observed in the adjacent normal mucosa [25]. Other investigators, however, maintain that the transition from normal colorectal mucosa to adenoma to cancer is associated with a progressive decline in stromal CD8+ T cell counts [10, 20, 28]. In light of our own data, we suspect that this latter trend might be more characteristic of nonpolypoid adenomas and that stable CD8+ cell densities across the normal mucosa-adenoma transition might be more typical of polypoid adenomas.

Stromal macrophages are key components of the tumor microenvironment that can influence tumor immunoediting (although their roles in the normal lamina propria of the gut are primarily phagocytic and bactericidal) [43]. In the stroma of our adenomas, macrophages (CD68+) were slightly more numerous than CD8+ or FOXP3+ T cells, but they were far less abundant than CD4+ T cells. Stromal macrophage densities were also significantly higher in polypoid lesions, although within the nonpolypoid group mild increases were observed with lesion size (**Fig 3**).

The functional heterogeneity and phenotypic plasticity of macrophage populations is even greater than that of other immune cell types. The two main polarization-based subtypes have more or less opposite functions in tumorigenesis: M1 macrophages are believed to exert anti-tumor effect by promoting the Th1 immune response; M2 macrophages favor the Th2 immune response, which facilitates tumor progression [44]. We used CD163 antibodies to quantitatively assess the latter tumor-promoting macrophage fraction in our colorectal adenomas. In both polypoid and nonpolypoid lesions, CD163+ cell densities were similar to those of CD68+ macrophages (**Fig 3**).

The predominance of M2 cells in the intratumoral macrophage population in these benign tumors was an unexpected finding, and it may not accurately reflect the characteristics of this early stage of tumorigenesis (i.e., presumably before the phase of tumor escape). CD163 is widely considered an M2-specific marker (reviewed in [15]), but Barros et al. [45] maintain that the macrophage polarization status can be more accurately classified on the basis of CD68 or CD163 expression plus transcription factor markers. Nevertheless, tumor infiltration by CD68+ macrophages tends to increase progressively as colorectal adenomas arise, grow, and become increasingly dysplastic ([23–25] and in our nonpolypoid lesions—**Fig 3**), and this trend seems to continue in the T1 and T2 stages of colorectal cancer [20]. However, immunohistochemistry studies by Edin et al. [19] indicate that colorectal cancer stage is inversely correlated with the densities of both M1 and M2 macrophages (identified by positivity for nitric oxide synthase 2 and CD163, respectively). Discordant findings have also emerged on the correlation between tumor-infiltrating macrophage densities and colorectal cancer prognosis or survival (reviewed in [15]).

MHC class I molecules are expressed by most nucleated cells. We used this marker only to obtain an overall count of stromal cells, a large proportion of which can reasonably be expected to be immune cells, including those not covered by other markers in our panel. Its nonspecificity for immune cells obviously precludes any speculations on the significance of our MHC-I+ cell counts in the process of immunoediting. Nonetheless, these counts provide complementary information on the picture that emerges based on expression patterns of the other 5 markers, i.e., that polypoid adenomas are characterized by more intense immune-cell stromal infiltration than their nonpolypoid counterparts. This difference might be related to the axial growth pattern of polypoid lesions, which protrude into the gut lumen. Compared with the lateral spread typical of nonpolypoid lesions, axial growth involves greater ramification and lengthening of the crypts. This type of growth is likely to require a more structured stromal support system, more replete with fibroblasts and vascular endothelial cells that would be reflected in higher MHC-I+ stromal cell counts.

The volume of a polypoid lesion is approximately twice that of a nonpolypoid with the same diameter, and the interstitial space available for immune / epithelial cell interaction is thus much larger. Early-stage polypoid colorectal cancers have more extensive microvascular networks than their nonpolypoid counterparts [46]. A similar difference might exist in precancerous lesions, and this facilitate the immune cells’ infiltration of the stroma of polypoid adenomas. The epithelial surface area might also play a role. Grivennikov et al. have suggested that the barrier function of adenomatous epithelium is compromised relative to that of the normal mucosa) [47]. If so, the larger surface area of a polypoid lesion (roughly twice that of a nonpolypoid adenoma with the same diameter) might result in a higher density of intralesional microbial antigens, which would trigger a more intense immune response. In our series and others, large nonpolypoid lesions (>10mm diameter) were more common in the proximal colon (**Table 4**), and tumor infiltration by immune cells might also be influenced by luminal factors peculiar to this colon segment. Serrated histology is also a common feature of nonpolypoid lesions, particularly those located in the proximal colon [48], but this factor had no effect on our findings since all but one of the lesions we studied were conventional adenomas (**Table 4**).

**Table 4:** Characteristics of the 40 colorectal adenomas included in the immunohistochemistry study.

Patient^a^	Age	Sex	Colorectal segment involved^b^	Maximum lesion diameter [mm]	Macroscopic appearance^c^	Dysplasia^d^
***Small polypoid lesions***						
1	37	M	S	16	*Ip*	low
2	74	M	D	15	*Ip*	low
3	74	M	S	16	*Ip*	high
4	58	M	T	15	*Ip*	low
5	63	F	S	15	*Ip*	low
6	60	F	S	15	*Is*	low
7	68	M	S	16	*Ip*	low
8	59	M	S	15	*Ip*	low
9	79	F	S	15	*Ip*	low
10	63	F	S	15	*Ip*	high
***Large polypoid lesions***						
11 *	68	F	S	30	*Ip*	low
12 *	83	M	S	40	*Ip*	low
13	72	F	S	30	*Ip*	high
14 *	64	M	T	50	*Ip*	low
15	57	F	S	35	*Ip*	high
16	84	M	S	35	*Is*	high
17	78	F	S	45	*Is*	high
18	88	M	S	30	*Is*	high
19	78	F	R	30	*Ip*	high
***Small nonpolypoid lesions***						
20	58	F	D	18	*IIa*	low
21	52	F	S	15	*IIa*	low
22	83	M	A	15	*IIa*	high
23	62	F	A	15	*IIa*	low
24	66	M	A	18	*IIa*	low
25	63	M	T	18	*IIa*	low
26	56	F	S	15	*IIa*	low
27	62	F	A	15	*IIa*	low
28	73	F	R	15	*IIa*	low
29	69	F	A	15	*IIa*	SSA
***Large nonpolypoid lesions***						
30	86	M	D	30	*IIa*	low
31	51	F	C	30	*IIa*	low
32	59	M	A	40	*IIa*	high
33	82	F	D	30	*IIa*	high
34	83	F	A	40	*IIa*	high
35	59	F	D	30	*IIa*	high
36	65	F	D	30	*IIa*	low
37	58	M	D	35	*IIa*	high
38	66	F	R	35	*IIa*	high
39	67	F	T	45	*IIa*	low
40	79	M	A	30	*IIa*	high

^a^ * CD4 staining was not available these lesions

^b^ Abbreviations: C, cecum; A, ascending colon; T, transverse; D, descending colon; S, sigmoid; R, rectum

^c^ Classified according to the Paris Endoscopic Classification of Superficial Neoplastic Lesions [29]

^d^ Highest degree of dysplasia in the lesion based on the WHO classification of tumors of the digestive system (Editorial and consensus conference in Lyon, France, November 6–9, 1999 [IARC]). SSA: sessile serrated adenoma.

## Conclusions

An obvious limitation of our study is the restricted panel of immune cell markers we used and the fact that each was investigated individually (i.e. one antibody per histologic section). The complexity of the immune landscape in these lesions would undoubtedly be captured more efficiently by less reductionist methods (e.g., recently developed high-throughput techniques, such as multicolor immunohistochemistry and immunofluorescence, multiparameter flow cytometry, and transcriptomic and proteomic analyses).

The differences we have documented between the immune infiltrates of precancerous colorectal tumors with different morphologies may have implications for tumor immuno-chemoprevention. For example, the ability of aspirin and other nonsteroidal anti-inflammatory agents to reduce the recurrence of colorectal adenomas (reviewed in [5]) has been attributed by some to these drugs’ lowering of prostaglandin E2 levels, whose production in colorectal tumors is governed by interactions between epithelial and stromal cells [41, 49]. Therefore, significant differences in the immune cell infiltrates found in the stroma of polypoid and nonpolypoid adenomas might cause these lesions to respond differently to this type of intervention.

## Materials and Methods

### Colorectal tissue samples

The first series of human colorectal tissues analyzed in this study has been extensively described in a previous report [50], which includes an overview of the transcriptomic changes occurring during the course of tumor development. It consisted of 42 precancerous lesions, each with a sample of normal mucosa removed from the same colon segment at a distance of >2 cm from the tumor. The tissues were prospectively collected during diagnostic colonoscopy and their transcriptomes analyzed with the GeneChip Human Exon 1.0 ST array (Affymetrix, Santa Clara, CA, USA). Raw microarray data are available in the Gene Expression Omnibus repository (Series GSE21962). Transcript levels were expressed as log2 lesion and normal mucosa ratios. For our analyses, the 42 lesions were classified according to endoscopic morphology (polypoid [type Ip] vs. nonpolypoid [type IIa]) and size (small [11–20 mm] vs. large [>20 mm]) [29].

The second series of samples was used for immunohistochemistry studies. It included FFPE blocks of 40 precancerous colorectal lesions from the Cremona Hospital Pathology Department archives (**Table 4**). One of the nonpolypoid lesions displayed the histologic features of a sessile serrated adenoma/polyp; the other 39 were adenomas with some degree of dysplasia. As expected, most of the nonpolypoid lesions came from the proximal colon (see Introduction). The marginal normal mucosa of each FFPE block was not suitable for cell counting due to cauterization artifacts. The tumors in this archival series were deliberately selected to produce two distinct, non-overlapping size classes: small (diameters measuring from 15 to <20 mm) and large (diameters of ≥30 mm). Intermediate size (20–29 mm) lesions were excluded to increase the chance of identifying biological differences between early (small) and more advanced (large) adenomas. Therefore, our size classes in these experiments are not based on the 10-mm cutoff commonly used to distinguish small polyps from advanced lesions requiring closer endoscopic follow-up [30]. Indeed, lesions measuring < 10 mm have been excluded from all of the studies we have conducted thus far to ensure that our sampling procedure had no negative effects on the histologic diagnosis.

Colorectal tumors with defective DNA mismatch repair are usually characterized by more abundant lymphocyte infiltrates than their mismatch repair-proficient counterparts. None of the patients whose tissues were examined in this study had family histories indicative of a predisposition to this type of tumor. In addition, none had ever been diagnosed with sporadic mismatch repair defective tumors, which are associated with transcriptional silencing of *MLH1*. DNA from the lesions we studied was not analyzed for colon cancer gene mutations.

Recruitment and analyses of both sample series were approved by the Ethics Committees of the Hospital of Cremona (Italy). Each donor provided written informed consent to collection and analysis of data and publication of the findings.

### Immunity-related transcriptomic (“immunome”) analysis (first series of 42 precancerous lesions)

Our analysis of transcriptomic data focused on the C7 immunologic signatures collection in the MSigDB, which is part of the Human Immunology Project Consortium (HIPC; http://www.immuneprofiling.org). GSEA was used to quantify immune-pathway upregulation or downregulation related to the morphology and size of the precancerous lesions. GSEA uses a variation of a Kolmogorov-Smirnov statistic to provide an enrichment score for each gene set. We use the signal-to-noise metric in the standard GSEA setting for measuring the correlation of a gene with the phenotype. The enrichment scores were then normalized to take into account the size of the gene sets resulting in a normalized enrichment score. This normalization was done using phenotypic permutations followed by standardization [39]. P-values and false discovery rates were computed using standard setting in the software. A p-value cut-off of 0.05 was used to define significant pathway enrichment.

### Tissue staining for immunologic markers (2nd series of 40 precancerous lesions)

Shortly after removal, tissues were fixed in neutral buffered formalin (pH 7.0), dehydrated, and embedded in paraffin using standard histological techniques. Sections from FFPE blocks were stained with hematoxylin-eosin and periodic acid-Schiff stain for routine histologic analysis. Serial sections (5-micron) were processed for immunohistochemistry on Leica BondMax instruments using Refine HRP-kits (Leica DS9800), including all buffer solutions from Leica Microsystems Newcastle. Antibody characteristics and immunostaining procedures are summarized in **Table 5**. A Reichert microscope with a digital camera (JTV Polyvar 2) and Sony Trinitron monitor were used for quantitative analysis of immunostained cells. Labelled cells were counted in 10 microscopic fields (each measuring 140 x 110 microns, total amplitude: 15,400 square microns) at 400X magnification.

**Table 5:** Antibodies used in the immunohistochemistry study.

Cell type	Cell Marker	Type	Antigen retrieval method *	Positive tissue control	Dilution	IHC detection protocol **	Supplier	Code	Isotype / Clone
T helper cells	**CD4**	Rabbit monoclonal	H2 20/95°C	Tonsil	1:100	rabbit HRP	Cell Marque Lifescreen	CMC10431021	IgG / SP35
cytotoxic T cells	**CD8**	Rabbit monoclonal	H2(40)	Tonsil	1:500	rabbit HRP	Cell Marque Lifescreen	CMC1083100	IgG / SP16
T regulatory	**FOXP3**	Rabbit monoclonal	H2(60)	Tonsil	1:200	rabbit HRP	Acris Antibodies	AM21067PU-M	IgG / SP97
macrophages	**CD68**	Mouse monoclonal	H2(30)	Tonsil	1:200	refine HRP	Novocastra Laboratories	NCL-L-CD68	IgG2a_Kappa / 514H12
macrophages	**CD163**	Mouse monoclonal	H2(30)	Tonsil	1:750	refine HRP	Serotec	MCA1853	IgG1 / EDHu-1
nucleolated cells	**MHC-I**	Rabbit monoclonal	H1(30)	Tonsil	1:500	rabbit HRP	Epitomics	2307–1	IgG / EPR1394Y

* Leica Bond Retrieval Buffer ER1 (30 min. at 100°C); Retrieval Buffer ER2 (for 30, 40 or 60 min at 100°C); Retrieval-Buffer ER2 for 20 min at 95°C.

** Rabbit HRP = Bond Polymer Refine HRP Kit from Leica (DS9800) without Postprimary Antibody (Rabbit anti-Mouse); Refine HRP: same Leica kit with Postprimary Antibody (Rabbit anti-Mouse)

MATLAB software (MathWorks, Natick, MA) was used for statistical analysis of the immunohistochemistry data. Non-parametric Wilcoxon Mann-Whitney tests were used to assess differences between mean values of immune cells in colorectal lesions according to their morphology, size, and degree of dysplasia. The significance level was set at p-value less than 0.05. MANOVA was used for testing the hypothesis that, given the sets of adenomatous lesions clustered in accordance to their morphology, size and degree of dysplasia, the means of the multiple response variable are or not distinct among the clusters of lesions, and the test is used to assess the statistical significance of the separation obtained among the lesions groups [51]. The null hypothesis H0 was that the two lesions groups came from the same population. MANOVA is accomplished by creating new variables (termed canonical) that maximize the differences among lesions of different morphology and/or size or degree of dysplasia. The first canonical variable is the linear combination of the original variables that best summarizes the differences among the lesion groups. The second canonical variable is the next best linear combination orthogonal to the first one, and so on. The grouped scatter plot of the first two canonical variables were used for displaying group structure in our data. Another graphical output of this analysis was the dendogram plot of the group mean clusters following MANOVA, where the clusters were computed by applying the single linkage method to the matrix of Mahalanobis distances between group means.

## Supporting Information

S1 Table Immune pathways displaying significant dysregulation in GSEA analysis of the five group comparisons. A total of 429 pathways (graphically represented in **Fig 1**) displayed significant up- or downregulation (p-value < 0.05) in GSEA of one or more of the five group comparisons: 1) polypoid vs. nonpolypoid (all sizes); 2) polypoid vs. nonpolypoid (small lesions only); 3) polypoid vs. nonpolypoid (large lesions only); 4) small vs. large (polypoid lesions only); 5) small vs. large (nonpolypoid lesions only). The column *degree* shows the number of group comparisons in which significant differential regulation of the pathway was found.
(XLSX)

## Abbreviations

GSEA: Gene Set Enrichment Analysis
MSigDB: Molecular Signature Database
FFPE: formalin-fixed paraffin-embedded
MANOVA: One-way Multivariate Analysis of Variance
T_reg_: T regulatory cells
